# Effectiveness and user experience of nose and throat swabbing techniques for SARS-CoV-2 detection: results from the UK COVID-19 National Testing Programme

**DOI:** 10.1186/s44263-024-00121-x

**Published:** 2025-01-13

**Authors:** Matthias E. Futschik, Raghavendran Kulasegaran-Shylini, Edward Blandford, Sean Harper, David Chapman, Elena Turek, Somya Agrawal, Valerie Phillips, Hannah Fordham, Lee Chan, Mike Kidd, Andrew Dodgson, Paul E. Klapper, Malur Sudhanva, Richard Vipond, Susan Hopkins, Tim Peto, Sarah Tunkel, Tom Fowler

**Affiliations:** 1 UK Health Security Agency, London, UK; 2 School of Biomedical Sciences, Faculty of Health, University of Plymouth, Plymouth, UK; 3 Deloitte MCS Ltd, London, UK; 4 School of Biological Sciences, University of Manchester, Manchester, UK; 5 Kings College Hospital NHS Foundation Trust, London, UK; 6 Health Protection Research Unit in Healthcare Associate Infections and Antimicrobial Resistance, National Institute for Health Research, Oxford, UK; 7 Nuffield Department of Medicine, University of Oxford, Oxford, UK; 8 William Harvey Research Institute and the Barts Cancer Institute, Queen Mary University of London, London, UK; 9 Public Health Wales, Cardiff, Wales UK

**Keywords:** Accessibility, Anterior nares, Assisted swabbing, COVID-19 diagnostics, Lateral flow devices, Mid-turbinate, Nose and throat, SARS-CoV2 diagnostics, Self-swabbing, Sensitivity

## Abstract

**Background:**

The UK’s National Health Service Test and Trace (NHSTT) program aimed to provide the most effective and accessible SARS-CoV-2 testing approach possible. Early user feedback indicated that there were accessibility issues associated with throat swabbing. We report the results of service evaluations performed by NHSTT to assess the effectiveness and user acceptance of swabbing approaches, as well as qualitative findings of user experiences from research reports, surveys, and incident reports. Our intent is to present and summarize our findings about the application of alternative swabbing approaches during the COVID-19 pandemic in the UK.

**Methods:**

From May 2020 to December 2021, NHSTT conducted a series of service evaluations assessing self-swabbing and assisted swabbing of the nose and throat, and nose only (anterior nares/mid-turbinate) using polymerase chain reaction (PCR) and lateral flow devices (LFDs), for diagnostic suitability within the COVID-19 National Testing Programme. Outcomes included observational user feedback on swabbing approaches and quantitative testing performance (concordance, sensitivity, and specificity). A post-hoc indirect comparison of swabbing approaches was also performed. Additionally, an analysis of existing cross-service research was conducted in April 2021 to determine user feedback regarding swabbing approaches.

**Results:**

Observational data from cross-service research indicated a user preference for nose swabbing over throat swabbing. Significantly more users reported that nose swabbing was easier to perform than throat swabbing (50% vs. 12%) and there were significantly fewer reported incidents. In the service evaluations, while there was reduced sensitivity for nose-only swabbing for PCR (88%) compared with nose and throat swabbing, similar sensitivities were observed for nose-only and nose and throat swabbing for LFDs. The sensitivity of nose-only swabbing for LFDs was higher for individuals with higher viral concentrations.

**Conclusions:**

User experience analyses supported a preference for nose-only swabbing. Nose-only swabbing for LFDs provided sufficient diagnostic accuracy, supporting its use as a viable option in the COVID-19 National Testing Programme. Less invasive swabbing approaches are important to maximize testing accessibility and alongside other behavioral interventions, increase user uptake.

**Supplementary Information:**

The online version contains supplementary material available at 10.1186/s44263-024-00121-x.

## Background

In response to the SARS-CoV-2 pandemic, the UK Government established the COVID-19 National Testing Programme and committed to mass testing, with initial testing commencing in March 2020 [1–3]. By May 2020, the program had evolved into the National Health Service Test and Trace (NHSTT) service with the objective to provide the most effective testing approach on a national scale [1, 4]. Accessibility of testing was one of the main focuses of the program and regional or local testing sites were rapidly established to allow SARS-CoV-2 testing [5]. Other initiatives to increase testing accessibility included exploring potentially easier sample collection approaches, such as the viability of saliva as a sampling method and whether remote support for testing could improve access to people who are blind and partially sighted [6].

In the initial period mainly molecular tests like quantitative reverse-transcription polymerase chain reaction (qRT-PCR, referred to as PCR hereafter), a highly sensitive and specific test that uses nucleic acid amplification to detect viral RNA [7–12] were used. While effective, PCR testing capacity was initially limited by the need for trained staff to undertake swabbing, a finite number of appointment slots, the need for individuals to attend an in-person testing site, high cost per test, and long turnaround times (24–72 h due to capacity). These limitations were overcome by the introduction of rapid antigen testing, which uses a point-of-care lateral flow device (LFD) to detect viral surface proteins produced during the active phase of SARS-CoV-2 infection [10]. Although not as sensitive as PCR, LFDs have several advantages as the results are usually available within 15–30 min and their low cost and convenience allow for a much higher volume of testing and broader accessibility [10, 13–16]. LFD tests were swiftly developed and evaluated for use in the UK by the late summer/autumn of 2020 [17–20]. PCRs were initially used in hospitals for the identification of cases, to prevent incursions of SARS-CoV-2 into healthcare settings, and for infection prevention and control in clinical settings. Outside of their use in clinical care, PCR testing was predominantly for testing symptomatic individuals for public health purposes (i.e., to identify if self-isolation and contact tracing were required). It was also used for asymptomatic testing with weekly PCR tests supplemented by further LFD testing. LFDs were initially used for asymptomatic mass testing and in research (SARS-CoV2 immunity and reinfection evaluation (SIREN) study) [21]. Over the course of the pandemic, LFD testing was expanded to symptomatic individuals to help people manage risk in assessing infectiousness to guide self-isolation timelines [22].

In the UK, performance requirements for PCR testing as part of the National COVID-19 Testing Programme were based on meeting the same standards as for clinical care [12]. For LFDs requirements for inclusion in the UK program were based on meeting laboratory performance evaluation criteria [17]. While the real-world performance for the first regulatory-approved self-test LFD [23] was generally considered a minimum benchmark, there was ongoing debate about the level of test performance that would be required to have a positive public health impact [24–26]. Specifically, questions arose regarding the importance of detecting infectious individuals (those with high viral concentrations) versus simply identifying infection [24, 26–28]. This created an urgent need to understand levels of performance that could be achieved to consider how LFDs may be effectively implemented.

The UK’s testing strategy and resultant policies were adapted and revised frequently by policymakers based on the changing epidemiology of the SARS-CoV-2 pandemic, updated scientific evidence, and the technology available at the time as well as socio- and economic factors [29–32]. As new diagnostic testing strategies became available and preferences for swabbing became apparent, NHSTT (with the Department of Health and Social Care [DHSC], later becoming part of the UK Health Security Agency) performed a series of service evaluations to assess the effectiveness and user acceptance of new testing and swabbing approaches, as well as to capture user experience and feedback. By analyzing both quantitative data from service evaluations and qualitative user feedback, we aim to provide insights into the most effective and accessible testing methods. The findings from this study are particularly relevant for informing testing strategies in future pandemics, where rapid and widespread testing will again be crucial.

## Methods

### NHSTT user attitude survey and analysis

The NHSTT LFD Product Research Team performed a post-hoc analysis to compare user preferences for nose and throat swabbing versus nose-only swabbing using existing cross-service user research as of April 2021. The existing research included testing surveys from service users and testing site leads, cross-service evaluation reports, and incident management reports related to swabbing. The goal was to see if nose-only swabbing would increase testing uptake. Data was analyzed in R (version 4.3.2) using prop.test, which implements a chi-squared test for equality of proportions with Yate’s continuity correction, to assess swab preferences and swabbing-related incidents.

Between March 4, 2021, and February 14, 2023, the Voice of the Customer (VOTC) program [16] captured feedback from SARS-CoV-2 testing experiences in England. Surveys were emailed to participants and data were collected regarding key touchpoints with the service: test, trace, and isolation. Responses were analyzed for common detractors, and the data was used to create a word cloud. Detractor proportions were calculated with R (version 4.3.2) using prop.test as above.

### Service evaluations and statistical analysis

The service evaluations reported here were part of the wider program conducted in the UK from May 2020 to December 2021 Fig. 1. Service evaluations compared swabbing techniques and testing performances, including if self-swabbing (SS) with LFDs at home was as effective as testing at a testing site. User feedback on swabbing experiences, where participants rated discomfort or pain during the process was collected as part of observational data. An overview of the service evaluations including start date, testing method, and swabbing technique can be found in Table 1.

**Fig. 1:**
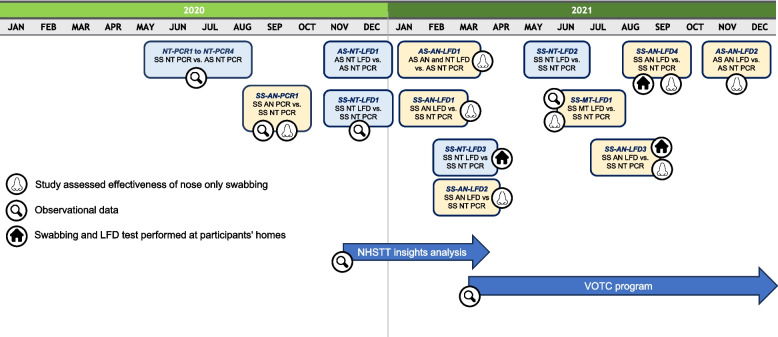
Timeline of key UK service evaluations*.* Figure is for illustration purposes, where the boxes are reflective of approximate study start date. *NT-PCR1* to *NT-PCR4*: AS versus SS of the nose and throat using PCR. *SS-AN-PCR1*: SS of the anterior nares using PCR. *AS-NT-LFD1*: AS of the nose and throat using the Innova LFD. *SS-NT-LFD1, SS-NT-LFD2*: SS of the nose and throat using the Innova LFD. *AS-AN-LFD1*: AS of nose and throat and anterior nares using the Orient Gene LFD. *SS-AN-LFD1*: SS of anterior nares using the Orient Gene LFD. *SS-AN-LFD2*: SS of anterior nares using the SureScreen LFD. *SS-NT-LFD3*: SS of the nose and throat using the Innova LFD at participants’ home. *SS-MT-LFD1*: SS of both nostrils to mid-turbinate level using the Innova LFD. *SS-AN-LFD3*: SS of anterior nares using the SureScreen LFD at participants’ home. *SS-AN-LFD4*: SS of anterior nares using the Innova LFD at participants’ home. *AS-AN-LFD*2: AS of anterior nares using Innova LFD. AN, anterior nares; AS, assisted swabbing; LFD, lateral flow device; MT, mid-turbinate level, NT, nose and throat; NHSTT, National Health Service Test and Trace; PCR, polymerase chain reaction; SS, self-swabbing; VOTC, Voice of the Customer

**Table 1:** Overview of the service evaluations

	Study start date	Test type	LFD test	Swabbing technique	Number recruited	Evaluable paired samples	Concordant samples, % (95% CI)	Sensitivity (vs. control), % (95% CI)		Specificity (vs. control), % (95% CI)
*Self-swabbing nose-only studies*										
* SS-AN-PCR1*	Sep 2020	SS PCR	N/A	Anterior nares	2169	2083	97.1 (96.3–97.8)	88.1 (84.4–91.2)		99.1 (98.5–99.5)
* SS-AN-LFD1* [20]	Jan 2021	SS LFD	Orient Gene	Anterior nares	1877	1788	89.4 (87.9–90.8)	53.3 (48.1–58.4)		99.3 (98.7–99.7)
* SS-AN-LFD2*	Feb 2021	SS LFD	SureScreen	Anterior nares	2524	2394	96.3 (95.5–97.0)	71.0 (65.5–76.0)		100.0 (99.8–100.0)
* SS-AN-LFD3*^a^	Jul 2021	SS LFD	SureScreen	Anterior nares	1534	1499	93.6 (92.2–94.8)	74.7 (69.9–79.1)		99.7 (99.1–99.9)
* SS-AN-LFD4*^a^	Aug 2021	SS LFD	Innova LFD	Anterior nares	6591	3990	95.3 (94.6–95.0)	74.9 (71.4–78.2)		99.2 (98.9–99.5)
* SS-MT-LFD1* [34]	Jun 2021	SS LFD	Innova LFD	Mid-turbinate	1156	1102	91.8 (90.0–93.4)	72.9 (67.7–77.7)		99.7 (99.1–100.0)
*Self-swabbing nose and throat studies*										
* SS-NT-LFD1* [18, 33, 35]	Nov 2020	SS LFD	Innova LFD	Nose and throat	2582	2473	90.6 (89.4–91.7)	50.0 (45.1–54.9)	99.1 (98.6–99.4)	
* SS-NT-LFD2* [34]	May 2021	SS LFD	Innova LFD	Nose and throat	635	631	65.8 (61.9–69.5)	65.8 (61.9–69.5)	N/A	
* SS-NT-LFD3*^a^	Feb 2021	SS LFD	Innova LFD	Nose and throat	957	773	95.2 (93.5–96.6)	56.8 (44.3–67.8)	99.7 (99.0–100.0)	
*Assisted swabbing studies*										
* AS-AN-LFD1* [20]	Jan 2021	AS LFD	Orient Gene	Anterior nares	2635	2597	92.8 (91.8–93.8)	53.2 (48.1–58.3)	99.8 (99.5–100.0)	
				Nose and throat	2635	2598	93.8 (92.8–94.7)	59.6 (54.6–64.6)	99.8 (99.5–99.9)	
* AS-AN-LFD2*	Nov 2021	AS LFD	Innova LFD	Anterior nares	1226	1160	89.2 (87.3–91.0)	57.6 (51.6–63.4)	99.4 (98.7–99.8)	
* AS-NT-LFD1* [18, 20, 33, 35]	Nov 2020	AS LFD	Innova LFD	Nose and throat	4356	4225	92.2 (91.3–93.0)	54.4 (50.6–58.2)	99.6 (99.3–99.8)	

*AN* anterior nares, *AS* assisted swabbing, *CI* confidence interval, *LFD* lateral flow device, *MT* mid-turbinate, *N/A* not applicable, *NT* nose and throat, *PCR* polymerase chain reaction, *qRT-PCR* quantitative reverse transcription polymerase chain reaction, *SS* self-swabbing

^a^Effectiveness of LFD tested at participants’ homes

Nose and throat sampling for qRT-PCR (either SS or AS) was the control for each service evaluation. Sensitivity = TP/(TP + FN) based on corresponding qRT-PCR test results; binomial 95% CIs, Pearson-Klopper method

Quantitative data was collected from various evaluations to compare the performance of the devices and swabbing approaches. Participants either swabbed their throat and nose or performed nose-only swabbing. For LFD tests, participants read and reported results themselves, while PCR samples were sent to laboratories. Sensitivity and specificity of LFDs were compared with PCR results, no specific performance benchmarks were set prior to the evaluations.

Matched LFD and qRT-PCR results were analyzed to determine concordance, sensitivity (stratified by viral concentration), and specificity. To calculate the 95% confidence intervals (CI) for sensitivity and specificity estimates, and observational data, the Clopper-Pearson exact method was used. Post-hoc analyses assessing the differences of proportions in the observational data were calculated in R (version 4.3.2) using prop.test.

Methodology and device performance data for some of these service evaluations have been partially reported elsewhere as part of the UK DHSC’s commitment to evaluating the performance of in-vitro diagnostic devices for use in the COVID-19 National Testing Programme [18, 20, 33–36].

Methods including specifics regarding the LFDs and PCR tests conducted in the service evaluations are detailed in the Additional file 1: Supplementary methods.

## Results

### User feedback on swabbing and testing approaches

#### NHSTT analysis of user preferences with LFD testing

From the testing surveys assessing swab preferences of the UK general public (*N* = 54,415) for PCR tests, significantly more participants found nose swabs easier to use compared with throat swabs (50% vs. 12%; *p* < 0.0001) (Additional file 1: Fig. S1A). From the cross-survey research reports, despite the small total number of reported incidents (*N* = 71), significantly more incidents of broken swabs were recorded through incident reporting with throat swabbing than with nose swabbing (*p* < 0.0001; Additional file 1: Fig. S1B).

#### VOTC user attitudes survey

Between March 4, 2021, and February 14, 2023, 1,289,611 entries were recorded in the VOTC survey regarding attitudes towards SARS-CoV-2 testing. Of these, 46,603 included the description of detractors of testing with 2473 entries including the word “swab” (Additional file 1: Fig. S2). Among these 29% included “throat” versus 16% included “nose”. The difference between the proportions was highly statistically significant (*p* < 0.0001) indicating a stronger negative association of throat swabbing compared with nose swabbing.

#### Observational data from the service evaluations

**Fig. 2:**
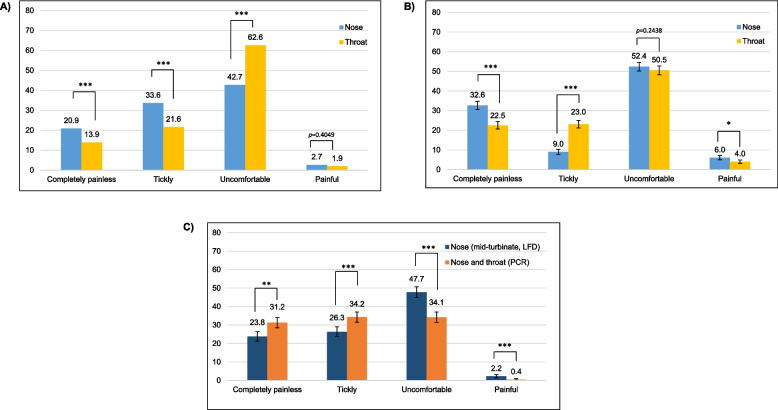
Observational user feedback and insight on experiences with swabbing approaches from seven UK service evaluation studies. **A** NT-PCR1 to NT-PCR4 (SS and AS of nose and throat). **B** SS-AN-PCR1 (SS anterior nares). **C** SS-MT-LFD1 (SS of both nostrils to mid-turbinate level)***.*** Data are mean and 95% CIs. *p* values derived from chi-sq tests comparing nose vs. throat swabbing. Blue bars represent nose-only swabbing (including nose-only and the nose part of nose and throat swabbing) for PCR. Yellow bars represent throat-only swabbing (the throat part of nose and throat swabbing) for PCR. Navy bars represent mid-turbinate nose swabbing for LFDs. Orange bars represent nose and throat swabbing for PCR. **A** *NT-PCR1* to *NT-PCR4*: AS vs. SS of the nose and throat for PCR. **B** *SS-AN-PCR1*: SS of the anterior nares for PCR vs. SS of nose and throat for PCR. **C** *SS-MT-LFD1*: SS of both nostrils to mid-turbinate level using the Innova LFD vs. SS of the nose and throat for PCR. *p < 0.005; **p < 0.0005; ***p < 0.0001. AN, anterior nares; AS, assisted swabbing; CI, confidence interval; LFD, lateral flow device; MT, mid-turbinate, NT, nose and throat; PCR, polymerase chain reaction; SS, self-swabbing

A total of 1392 participants from studies *NT-PCR1* to *NT-PCR4* provided feedback on their experience with nose and throat swabbing. Significantly greater proportions of participants found throat swabbing to be more uncomfortable than nose swabbing (63% vs. 43%), and nose swabbing to be completely painless compared with throat swabbing (21% vs. 14%) (*p* < 0.0001; Fig. 2A). In study *SS-AN-PCR1* (*n* = 1962), similar proportions of participants found nose swabbing (anterior nares, swallow nose) and throat swabbing to be uncomfortable (52% vs. 51%), but significantly greater proportions of participants found nose swabbing to be completely painless (33% vs. 23%; *p* < 0.0001) (Fig. 2B). In contrast, significantly greater proportions of patients reported that mid-turbinate (deeper nose) swabbing was uncomfortable compared with nose and throat swabbing (48% vs. 34%; *p* < 0.0001) in study *SS-MT-LFD1* (*n* = 1148) (Fig. 2C).

### Service evaluations: quantitative testing outcomes

An overview of the number of participants recruited, the paired samples evaluable for analysis, and the quantitative testing outcomes in the service evaluations are shown in Table 1. Numbers of void samples and other omitted samples are shown in Additional file 1: Table S2. Key findings of the service evaluations and subsequent indirect comparison are reported below according to the different research questions covered in this report.

#### Can shallow nose swabbing provide the same performance as nose and throat swabbing using PCR tests?

**Fig. 3:**
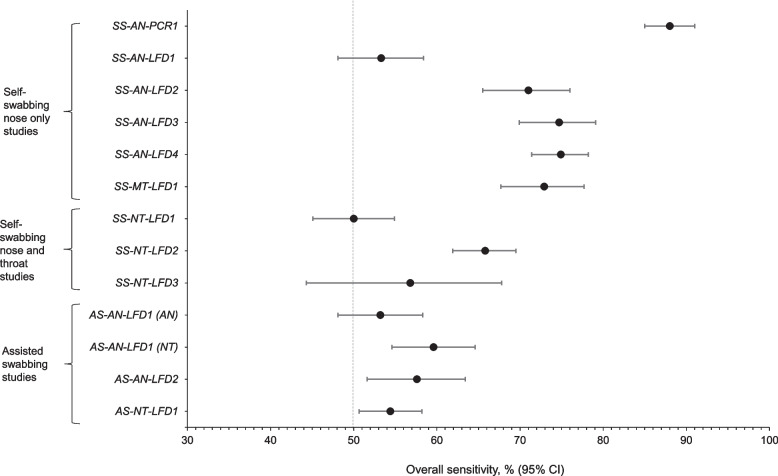
Overall point sensitivities of key UK service evaluations*. SS-AN-PCR1*: SS of the anterior nares using PCR. *SS-AN-LFD1*: SS of anterior nares using the Orient Gene LFD. *SS-AN-LFD2*: SS of anterior nares using the SureScreen LFD. *SS-AN-LFD3*: SS of anterior nares using the SureScreen LFD at participants’ home. *SS-AN-LFD4*: SS of anterior nares using the Innova LFD at participants’ home. *SS-NT-LFD1, SS-NT-LFD2*: SS of the nose and throat using the Innova LFD. *SS-NT-LFD3*: SS of the nose and throat using the Innova LFD at participants’ home. *AS-NT-LFD1*: AS of the nose and throat using the Innova LFD. *AS-AN-LFD1*: AS of nose and throat and anterior nares using the Orient Gene LFD. *SS-MT-LFD1*: SS of both nostrils to mid-turbinate level using the Innova LFD. *AS-AN-LFD*2: AS of anterior nares using Innova LFD. Sensitivity = TP/(TP + FN) based on corresponding qRT-PCR test results; binomial 95% CIs, Pearson-Klopper method. AN, anterior nares; AS, assisted swabbing; CI, confidence interval; FN, false negative; LFD, lateral flow device; MT, mid-turbinate level, NT, nose and throat; PCR, polymerase chain reaction; SS, self-swabbing; TP, true positive. The dotted vertical line indicates a diagnostic sensitivity of 50%, which was deemed sufficient in a real-world setting for use in the NHSTT program

Study *SS-AN-PCR1* evaluated the effectiveness of self-swabbing (SS) of the anterior nares (shallow nose) for identifying SARS-CoV-2 compared with SS of the nose and throat for PCR. Of the 2083 paired PCR samples evaluable, 97.1% were concordant for positive (*n* = 336) or negative (*n* = 1688) results. Of the 59 discordant samples, 46 were positive in the nose and throat samples only (Additional file 1: Table S3). There was a statistically significant difference between discordant results in favor of nose and throat swabbing versus nose-only swabbing (*p* < 0.001). The overall sensitivity of SS of the anterior nares for PCR was 88% (Table 1; Fig. 3).

Analysis of 109 paired samples classified into differing viral concentration groups based on PCR cycle threshold value (Ct), 87 showed higher concentrations for nose and throat swabbing and 38 showed higher concentrations for nose-only swabbing (*p* < 0.001) (Additional file 1: Table S4). Of the 46 samples which were positive for nose and throat swabbing and negative for nose-only swabbing, 18 had a Ct value of < 25 (higher viral concentration > 10,000 (10 K) viral copies/mL).

**Fig. 4:**
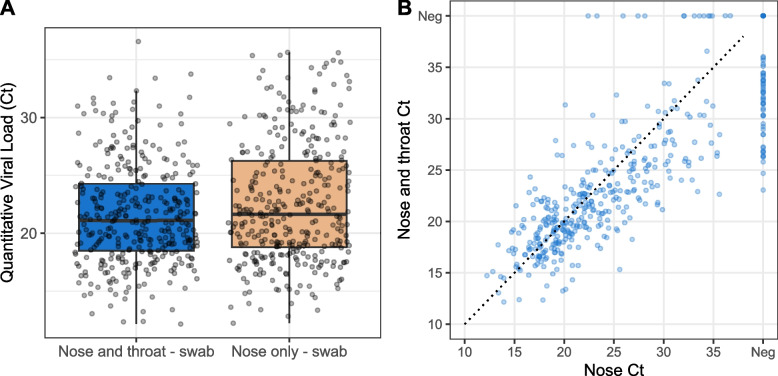
Comparison of SARS-CoV-2 viral concentration in SS-AN-PCR1. **A** Average Ct of nose and throat versus nose only swabs in SARS-CoV-2–positive individuals. **B** Correlation of Ct between nose and throat versus nose only swabs in all individuals***.**** SS-AN-PCR1*: SS of the anterior nares using PCR. **A** The average Ct value was taken across the three genes (ORF1ab gene, N gene, and S-gene) in all concordant and discordant SARS-CoV-2–positive individuals; the boxplot shows a comparison between the SS and AS samples. There was a statistically significant difference in Ct values for nose and throat swabs versus nose-only swabs: mean − 0.99; 95% CI − 1.3, − 0.6; *p* < 0.001. **B** There was a strong correlation (Spearman’s rho correlation co-efficient = 0.87; *p* < 0.001) between the average Ct values of the nose and throat, and nose-only samples. AN, anterior nares; CI, confidence interval; Ct, cycle threshold; NT, nose and throat; PCR, polymerase chain reaction; SS, self-swabbing

The average Ct score for nose and throat swabbing was 21.6 (standard error [SEM] 0.24) and for nose-only swabbing was 22.6 (SEM 0.28), indicating a lower viral concentration with anterior nares swabbing (*p* < 0.001; *t*-test). There was a strong correlation (Spearman correlation *r* = 0.87, *p* < 0.001) between the average Ct scores of the nose and throat, and nose-only samples (Fig. 4).

Overall, *SS-AN-PCR1* demonstrated that SS of anterior nares has reduced sensitivity (88%) versus nose and throat swabbing when used for PCR testing as less viral material tended to be collected.

#### Can nose swabbing (via anterior nares or mid-turbinate level) provide the same diagnostic performance as nose and throat swabbing using LFDs?

##### Assisted swabbing of anterior nares

Study *AS-AN-LFD1* assessed whether assisted swabbing (AS) of both nose and throat and double anterior nares techniques using the Orient Gene COVID-19 Ag Rapid Test Cassette provided sufficient diagnostic performance. For the phase of the study assessing swabbing of the anterior nares, there were 2597 evaluable paired PCR and LFD samples, of which 92.8% were concordant (*n* = 207 positive and *n* = 2204 negative) (Additional file 1: Table S5). Overall sensitivity for anterior nares swabbing was 53.2%, with a higher sensitivity of 88.3% for samples with high viral concentration (> 1 million (1 M) viral copies/mL). Overall sensitivity was 53.2%, with a higher sensitivity of 88.3% for samples with high viral concentration (> 1 M copies/mL), (Additional file 1: Table S6) [20]. Assisted swabbing of the nose and throat had a non-statistically higher sensitivity (59.6%) compared to double anterior nares (53.2%) (Table 1, Fig. 3, Additional file 1: Table S6).

Study *AS-AN-LFD2* investigated the effectiveness and suitability of AS of both anterior nares using the Innova LFD test. Of the 1160 evaluable paired PCR and LFD tests, 89.2% were concordant for positive (*n* = 163) or negative results (*n* = 872) (Additional file 1: Table S5). Overall sensitivity was 57.6% with a higher sensitivity of 89.5% for samples with high viral concentration (> 1 M copies/mL; Additional file 1: Table S6). Comparison of overall sensitivity in *AS-AN-LFD2* (anterior nares) and *AS-NT-LFD1* (nose and throat [35]) suggested a similar sensitivity overall (57.6% vs. 54.4%) with overlapping 95% CIs indicating similar performance depending on viral concentration (Table 1, Fig. 3, Additional file 1: Table S6).

##### Self-swabbing of anterior nares and/or mid-turbinate level

Study *SS-MT-LFD1* evaluated the effectiveness of SS of both nostrils to mid-turbinate level (deeper nose) using the Innova LFD test. A total of 91.8% of participants with paired PCR and LFD samples were concordant for a positive (*n* = 237) or negative (*n* = 775) result (Additional file 1: Table S7). Overall sensitivity for LFD double nose (to mid-turbinate level) swabbing was 72.9% and was higher (98.1%) for samples with viral concentration > 1 M copies/mL (Additional file 1: Table S8) [34]. Indirect comparison of overall sensitivity observed in studies *SS-MT-LFD1* and *SS-NT-LFD2* [34], suggested significantly greater sensitivity for *SS-MT-LFD1* compared to *SS-NT-LFD2* (Table 2).

**Table 2:** Indirect comparison of sensitivity of different self-swabbing approaches (mid-turbinate vs. nose and throat)

	*SS-MT-LFD1*^a^ [34] Mid-turbinate (N = 1102)						*SS-NT-LFD2*^c^ [34] Nose and throat (*N* = 635)						Difference (95% CI); *p* value^d^
	*N*	TP	TN	FP	FN	Sensitivity, % (95% CI)^b^	*N*	TP	TN	FP	FN	Sensitivity, % (95% CI)^b^	
Overall	1102	237	775	2	88	72.9 (67.7–77.7)	631	415	0	0	216	65.8 (61.9–69.5)	7.2 (0.8–13.5); *p* = 0.03
Viral concentration													
< 10 K	48	9	0	0	39	18.8 (9.0–32.6)	66	13	0	0	53	19.7 (10.9–31.3)	− 1.0 (− 16.5–14.6); *p* = 1.00
10 K–1 M	110	70	0	0	40	63.6 (53.9–72.6)	216	118	0	0	98	54.6 (47.7–61.4)	9.0 (− 2.9–20.9) *p* = 0.15
> 1 M	154	151	0	0	3	98.1 (94.4–99.6)	349	284	0	0	65	81.4 (76.9–85.3)	16.7 (11.6–21.8) *p* < 0.005

*CI* confidence interval, *FN* false negative, *FP* false positive, *LFD* lateral flow device, *MT* mid-turbinate, *NT* nose and throat, *PCR* polymerase chain reaction, *SS* self-swabbing, *TN* true negative, *TP* true positive

*SS-MT-LFD1* SS of both nostrils to mid-turbinate level with the Innova LFD. *SS-NT-LFD2* SS of the nose and throat with the Innova LFD

^a^Does not include any samples that were missing, void, or dropped out for PCR and LFD

^b^Sensitivity = TP/(TP + FN), Binomial 95% *CIs* Pearson-Klopper method

^c^Dataset for final analysis only contained PCR–positive samples

^d^Two-sample Chi-squared test for equality of proportions, comparing the sensitivity between studies *SS-MT-LFD1* versus *SS-NT-LFD2*

Two further studies, *SS-AN-LFD1* (Orient Gene COVID-19 Ag Rapid Test Cassette) and *SS-AN-LFD2* (SureScreen LFD) assessed the effectiveness of SS of the anterior nares using LFDs (Table 1, Fig. 3, Additional file 1: Tables S7 and S8). For the study, *SS-AN-LFD1*, 89.4% of samples were concordant for positive (*n* = 204) or negative (*n* = 1395) results. Sensitivity was 53.3% overall, and 87.8% for samples with viral concentration > 1 M copies/mL [20]. *SS-AN-LFD2* had similar results with the SureScreen LFD, reporting an overall sensitivity of 71.0%, which was higher (95.2%) for samples with viral concentration > 1 M copies/mL.

These studies demonstrated that SS and AS of the anterior nares and SS of both nostrils to mid-turbinate level for LFDs provide sufficient diagnostic sensitivity which was comparable to nose and throat swabbing for the same LFD test. SS of both nostrils to mid-turbinate level had greater sensitivity than SS of the nose and throat for the Innova LFD.

#### Can self-swabbing and sampling at users’ homes provide the same diagnostic accuracy as self-testing at a dedicated testing site?

##### Self-swabbing of the nose and throat

Study *SS-NT-LFD3* explored whether participant-reported performance using the Innova LFD (via SS of nose and throat at home) provided sufficient diagnostic accuracy and was comparable to that at a test site. Of the 773 evaluable paired samples, 46 were concordant for a positive result and 690 for a negative result (Additional file 1: Table S9). Overall sensitivity and specificity of the Innova LFD at home were 56.8% and 99.7%, respectively (Table 1). Sensitivity was higher (90.6%) for viral concentrations > 1 M copies/mL (Additional file 1: Table S10). The performance of the Innova LFD when used at home was similar to that in *SS-NT-LFD1* [33, 35] (Table 1, Fig. 3, Additional file 1: Table S7) when the sample was taken at a dedicated test site, based on overlapping 95% CIs.

##### Self-swabbing of the anterior nares

Studies *SS-AN-LFD3* and *SS-AN-LFD4* assessed the effectiveness of SS of the anterior nares with the SureScreen LFD and Innova LFD, respectively, in a home setting. A total of 93.6% paired PCR and LFD samples were concordant in *SS-AN-LFD3* (Additional file 1: Table S9). Sensitivity was 74.7% overall (Table 1, Fig. 3) and 93.3% for samples with viral concentration > 1 M copies/mL (Additional file 1: Table S10). Comparison of the sensitivity of the SureScreen LFD test in the *SS-AN-LFD3* (home setting) and *SS-AN-LFD2* (test site) studies suggested a similar sensitivity overall with overlapping 95% CIs (Table 1, Fig. 3).

*SS-AN-LFD4* had an overall concordance of 95.3% (Additional file 1: Table S9) with a sensitivity of 74.9% and specificity of 99.2% (Table 1, Fig. 3). Sensitivity was higher (97.6%) at higher viral concentration (Additional file 1: Table S10). Comparison of sensitivity of SS with LFDs in studies *SS-AN-LFD4* (Innova LFD at home, anterior nares) *SS-NT-LFD1* (Innova, nose and throat), *SS-NT-LFD3* (Innova, nose and throat), and *SS-AN-LFD1* (Orient Gene, anterior nares) suggested that the at-home anterior nares testing approach used in *SS-AN-LFD4* had better sensitivity (both overall and at high viral concentration) and more likely to correctly predict a positive result than the approaches used in studies *SS-NT-LFD1*, *SS-NT-LFD3*, and *SS-AN-LFD1* (Table 3).

**Table 3:** Pairwise comparison of the sensitivity of self-swabbing of anterior nares vs nose and throat

Study	*N*	TP	FN	*p* value
Overall				
* SS-AN-LFD4* Anterior nares	649	486	163	
* SS-NT-LFD1* Nose and throat	424	212	212	< 0.001
* SS-NT-LFD3* Nose and throat	632	416	216	< 0.001
* SS-AN-LFD1* Anterior nares	383	204	179	< 0.001
*Viral concentration* > *1 M*				
* SS-AN-LFD4* Anterior nares	286	279	7	
* SS-NT-LFD1* Nose and throat	118	95	23	< 0.001
* SS-NT-LFD3* Nose and throat	375	305	70	< 0.001
* SS-AN-LFD1* Anterior nares	139	122	17	< 0.001
*Viral concentration* > *10 K–1 M*				
* SS-AN-LFD4* Anterior nares	229	172	57	
* SS-NT-LFD1* Nose and throat	189	103	86	< 0.001
* SS-NT-LFD3* Nose and throat	207	113	94	< 0.001
* SS-AN-LFD1* Anterior nares	131	70	61	< 0.001
Viral concentration < 10 K				
* SS-AN-LFD4* Anterior nares	122	27	95	
* SS-NT-LFD1* Nose and throat	117	14	103	0.037
* SS-NT-LFD3* Nose and throat	45	9	36	0.767
* SS-AN-LFD1* Anterior nares	113	12	101	0.018

*p* values derived from chi-sq tests comparing overall and viral concentration sensitivities of *SS-AN-LFD4* with the other service evaluations

*SS-AN-LFD4* SS of the anterior nares with the Innova LFD at participants’ homes (reference). *SS-NT-LFD1* SS of the nose and throat with the Innova LFD*. SS-NT-LFD3* SS of the nose and throat with the Innova LFD. *SS-AN-LFD1* SS of the anterior nares with the Orient Gene LFD

*AN* anterior nares, *CI* confidence interval, *FN* false negative, *LFD* lateral flow device, *NT* nose and throat, *SS* self-swabbing, *TP* true positive

Overall, these findings demonstrated that self-swabbing at home was as effective as swabbing at dedicated test sites for testing with LFDs.

## Discussion

Throughout the SARS-CoV-2 pandemic, NHSTT aimed to provide the most effective testing approach on a national scale, by increasing the availability of SARS-CoV-2 tests and the speed of the testing process [1]. From the beginning of the program, it was recognized that the more invasive the swabbing approach, the more of a challenge sample collection would be in increasing test-seeking behavior and test accessibility. For the majority of the pandemic when symptomatic PCR testing was used, a negative test result had a direct impact on the individual, as the individual and their contacts did not need to continue self-isolating; therefore, identifying alternative approaches for those who could not complete a nose and throat swab was also an important element of accessibility. For regular twice-a-week asymptomatic LFD testing, factors likely to impact ongoing compliance were also key [37, 38]. Consistent user feedback, as demonstrated in the service evaluations and survey research reported here, indicated that there was a preference for less invasive approaches such as nose-only swabbing instead of nose and throat swabbing. In particular, participants reported operational difficulties with throat swabbing such as identifying the tonsils, gagging and sickness, and incidents of broken swabs. The observational data from the service evaluations supported the preference for nose over throat swabbing. The feedback on nose-only swabbing showed that the location and depth of swabbing impacted user experience. A preference was reported for shallow nose (anterior nares) swabbing, with feedback from study *SS-MT-LFD1* reporting some negative experiences with (deeper nose) mid-turbinate.

Demonstration of the acceptable use of nose-only swabbing was expected to have several benefits, including increased tolerability of swabbing and increased test-seeking behavior, decreased risk of harm associated with throat swabbing, and alignment of instructions for users of different LFDs. However, without understanding the effect of the different swabbing methods, informed decision-making on UK policy could not be implemented. To this end, the program of service evaluation studies performed by NHSTT during the SARS-CoV-2 pandemic included those that evaluated the effectiveness and user acceptance of new testing and swabbing approaches (e.g., nose-only swabbing to the mid-turbinate level or anterior nares). The results subsequently reported here helped to inform decision-making and UK policy regarding SARS-CoV-2 testing approaches and techniques and supported the decision for nose-only LFD swabbing to be included in the COVID-19 National Testing Programme [6, 29].

Importantly, validation of different swabbing approaches needed to be considered against the potential impact of reduced sensitivity and false negatives, as well as self-isolation requirements [39]. SARS-CoV-2 testing in the UK was initially rolled out through a population-specific service delivery model comprising PCR testing with nose and throat swabbing for symptomatic individuals at dedicated test sites [5, 11]. Findings from Study *SS-AN-PCR1* demonstrated a reduced sensitivity (of ~ 10%) with PCR analysis for nose-only swabbing compared with nose and throat swabbing, which prevented its widespread use in the context of symptomatic PCR testing. However, nose-only swabbing with PCR was still deemed adequate for public health testing as a viable alternative for those who could not perform nose and throat swabbing [40, 41]. For example, the use of nose-only swabbing with PCR could allow key workers who were required to self-isolate, but who could not perform nose and throat swabbing, to test and return to work if they had a negative PCR result when they were otherwise required to remain in self-isolation [42, 43].

From April 2021, LFDs were made freely and universally available in the UK as part of mass community testing, and asymptomatic individuals were encouraged to test twice weekly to identify SARS-CoV-2-positive cases and further reduce community transmission [16]. Findings from the initial *AS-NT-LFD1* and *SS-NT-LFD1* studies supported that swabbing of the nose and throat with LFDs provided sufficient diagnostic sensitivity (at least 50%) in a real-world setting for use in the NHSTT program [17–20, 33–35]. A sensitivity threshold of 50% was initially deemed acceptable for the use of LFDs and formed the basis for the original authorizations granted by the Medicines and Healthcare Products Regulatory Agency for the first self-test LFD [23]. Sensitivity was higher for high viral concentration, which is associated with increased infectiousness [17, 18, 27, 44], which allowed the rollout of population-wide asymptomatic testing with LFDs. Several subsequent service evaluations (studies *AS-AN-LFD1*, *AS-AN-LFD2*, *SS-AN-LFD1* to *SS-AN-LFD4*, and *SS-MT-LFD1*) demonstrated a similar sensitivity of LFD tests to identify SARS-CoV-2 when using nose only (anterior nares [shallow nose] and mid-turbinate [deeper nose]) swabbing compared with nose and throat, supporting the use of nose only swabbing as a viable alternative and further increasing access to testing. The lower sensitivities of LFDs for identifying SARS-CoV-2 compared with PCR are mitigated by the different testing purposes for which they were predominantly used, with a likely increase in testing uptake and repeated testing [16, 45]. Modeling work has shown that repeated testing can contribute to a reduction in transmission [15, 46].

Across the service evaluation studies, using PCR and LFD, nose-only swabbing showed a high sensitivity for identifying SARS-CoV-2 among individuals with high viral concentrations (> 1 M copies/mL). The ability of nose-only swabbing to identify individuals with a high viral concentration is valuable because such individuals are most likely to be infectious [36, 41, 47]. For example, one study demonstrated that less than half of PCR-positive asymptomatic individuals were shedding SARS-CoV-2 whereas LFDs had more than 80% sensitivity in detecting those shedding SARS-CoV-2 [48].

While there was a reported preference for nose-only swabbing compared with nose and throat swabbing, the post-hoc analysis conducted by NHSTT did highlight that some uncertainty exists among test users regarding how far to insert the swab within the nostrils. While this is not a major concern in terms of barriers to testing accessibility, further research or supportive guidance on this issue may be appropriate in the future. Furthermore, the impact of the user swabbing experience on adherence and uptake was not assessed in the service evaluations. However, it is plausible to assume that the reported discomfort of throat swabbing may act as a deterrent to testing and that less invasive techniques, such as nose-only swabbing, may encourage test-seeking behavior.

It is important to note that the service evaluations were not designed to directly compare differences in diagnostic performance between swabbing approaches, but rather performance with respect to a chosen reference swabbing approach, and thus the cross-study comparisons presented here were exploratory and post-hoc. Changes in confounding variables might have an impact on the results and their robustness. In particular, the series of service evaluations assessed various LFDs and swabbing approaches and were conducted at different stages of the pandemic, and thus potential confounding with other variables (e.g., variants and vaccination) might exist in the comparison across service evaluation studies. Service evaluations did not compare directly to benchmarked standards for different use cases, such as symptomatic and asymptomatic mass testing, though evaluations of the testing program suggest performance was adequate to have a substantial public health impact [4]. Participation used a convenience sampling method of only asking site attendees to be tested, and thus study populations were not necessarily representative of the general population, but instead of those able and willing to attend regional and local testing sites. A similar caution is warranted in the interpretation of the user survey data and their representativeness, these were developed as part of an operational response and conformed with best practice for quality feedback. Finally, the data collection period ended in December 2021, prior to the dominance of the Omicron variants in the UK. Given the differences between the Omicron variants and earlier strains, the findings regarding the diagnostic accuracy of various swabbing techniques may not fully apply to the current situation.

In response to future pandemics, there need to be preliminary investigations assessing test and sample development to maximize testing accessibility and usability among users and encourage test-seeking behavior [49–53]. For any pandemic requiring mass community testing, the invasiveness of a swabbing approach is always likely to be a barrier to testing [53]. There may be instances where the sample collection and testing approach interfere with the accessibility to testing, and there may be other scenarios where the approach is less relevant to the individual but may affect the overarching intervention [52, 53]. The work reported here in the context of the SARS-CoV-2 pandemic demonstrates that mass community testing can be conducted using less invasive techniques. Although further validation is required, it is not unreasonable to assume that the results can be generalized for future scenarios and would be a basis for implementation, at least in the early stages while further validation is undertaken. Importantly, we demonstrated that testing performance is not affected by whether individuals test at home or at a testing site which further improves accessibility to testing [4, 33, 54].

## Conclusions

User experience analyses performed in the UK during the SARS-CoV-2 pandemic supported a preference for nose-only swabbing compared with nose and throat swabbing. The findings from the service evaluation studies performed by NHSTT demonstrated the acceptable diagnostic performance of nose-only swabbing for LFDs in a real-world setting and supported the use of nose-only swabbing (for LFDs) as a viable option for use in the UK national testing initiative. The results of these studies informed and supported policy decisions regarding SARS-CoV-2 testing in the UK during the early phase of the SARS-CoV-2 pandemic and emphasized the importance of less invasive testing approaches to maximize testing accessibility in future pandemic responses.

## Supplementary Information

Additional file 1: Supplementary methods, Supplementary results, Table S1: Study ID of service evaluations, Table S2: Numbers of void samples and other omitted samples in the service evaluations, Table S3: SS-AN-PCR1: Concordance matrix of nose and throat swabbing versus nose only (anterior nares) swabbing, Table S4: SS-AN-PCR1 (anterior nares): quantitative data analysis by Ct. Table S5: Concordance matrix of LFD and PCR results in assisted-swabbing studies: AS-NT-LFD1 (nose and throat), AS-AN-LFD1 (anterior nares), and AS-AN-LFD2 (anterior nares). Table S6: Sensitivity of assisted swabbing using LFDs overall and by viral concentration, compared with assisted swabbing of the nose and throat with PCR, in studies AS-NT-LFD1 (nose and throat), AS-AN-LFD1 (anterior nares), and AS-AN-LFD2 (anterior nares). Table S7: Concordance matrix of LFD and PCR results in Studies SS-NT-LFD1 (nose and throat), SS-NT-LFD2 (nose and throat), SS-AN-LFD1 (anterior nares), SS-AN-LFD2 (anterior nares), and SS-MT-LFD1 (mid-turbinate level). Table S8: Sensitivity of self-swabbing using LFDs overall and by viral concentration, compared with self-swabbing of the nose and throat with PCR, in Studies SS-NT-LFD1 (nose and throat), SS-AN-LFD1 (anterior nares), SS-AN-LFD2 (anterior nares), SS-NT-LFD2 (nose and throat), and SS-MT-LFD1 (mid-turbinate level). Table S9: Concordance matrix of LFD and PCR results in Studies SS-NT-LFD3 (nose and throat), SS-AN-LFD3 (anterior nares), and SS-AN-LFD4 (anterior nares). Table S10: Sensitivity of at-home self-swabbing using LFDs overall and by viral concentration, compared with self-swabbing of the nose and throat with PCR, in Studies SS-NT-LFD3 (nose and throat), SS-AN-LFD3 (anterior nares), and SS-AN-LFD4 (anterior nares). Fig. S1: User experience findings from the LFD Product Research Team. A Ease of use with nose and throat swabbing (*N*=54,415). B Recorded incidents of broken swabs with nose and throat swabbing (PCR and LFD). Fig. S2 Word cloud summary of detractor descriptions for SARS-CoV-2 swabbing.

## Data Availability

The datasets generated and/or analyzed during the current study are not publicly available as they were collected as part of a public health response and are considered sensitive. Access to anonymized data will be considered by the corresponding author on reasonable request.

## Abbreviations

AN: Anterior nares
AS: Assisted swabbing
CI: Confidence interval
Ct: Cycle threshold
DHSC: Department of Health and Social Care
FN: False negative
FP: False positive
HRA: Health Research Authority
LFD: Lateral flow device
MT: Mid-turbinate
N/A: Not applicable
NHSTT: National Health Service Test and Trace
NT: Nose and throat
PCR: Polymerase chain reaction
PHE REGG: Public Health England’s Research Ethics and Governance Group
qRT-PCR: Quantitative reverse transcription-polymerase chain reaction
SEM: Standard error
SS: Self-swabbing
TN: True negative
TP: True positive
UKHSA: UK Health Security Agency
VOC: Variant of concern
VOTC: Voice of the Customer
