# Supplementary material for: Effectiveness and user experience of nose and throat swabbing techniques for SARS-CoV-2 detection: results from the UK COVID-19 National Testing Programme
Source: BMC Glob Public Health. 2025 Jan 13;3:5. doi: 10.1186/s44263-024-00121-x (PMC11731392; doi:10.1186/s44263-024-00121-x)
Supplement: Supplementary file 1 — Additional file 1: Supplementary methods, Supplementary results, Table S1: Study ID of service evaluations, Table S2: Numbers of void samples and other omitted samples in the service evaluations, Table S3: SS-AN-PCR1: Concordance matrix of nose and throat swabbing versus nose only (anterior nares) swabbing, Table S4: SS-AN-PCR1 (anterior nares): quantitative data analysis by Ct. Table S5: Concordance matrix of LFD and PCR results in assisted-swabbing studies: AS-NT-LFD1 (nose and throat), AS-AN-LFD1 (anterior nares), and AS-AN-LFD2 (anterior nares). Table S6: Sensitivity of assisted swabbing using LFDs overall and by viral concentration, compared with assisted swabbing of the nose and throat with PCR, in studies AS-NT-LFD1 (nose and throat), AS-AN-LFD1 (anterior nares), and AS-AN-LFD2 (anterior nares). Table S7: Concordance matrix of LFD and PCR results in Studies SS-NT-LFD1 (nose and throat), SS-NT-LFD2 (nose and throat), SS-AN-LFD1 (anterior nares), SS-AN-LFD2 (anterior nares), and SS-MT-LFD1 (mid-turbinate level). Table S8: Sensitivity of self-swabbing using LFDs overall and by viral concentration, compared with self-swabbing of the nose and throat with PCR, in Studies SS-NT-LFD1 (nose and throat), SS-AN-LFD1 (anterior nares), SS-AN-LFD2 (anterior nares), SS-NT-LFD2 (nose and throat), and SS-MT-LFD1 (mid-turbinate level). Table S9: Concordance matrix of LFD and PCR results in Studies SS-NT-LFD3 (nose and throat), SS-AN-LFD3 (anterior nares), and SS-AN-LFD4 (anterior nares). Table S10: Sensitivity of at-home self-swabbing using LFDs overall and by viral concentration, compared with self-swabbing of the nose and throat with PCR, in Studies SS-NT-LFD3 (nose and throat), SS-AN-LFD3 (anterior nares), and SS-AN-LFD4 (anterior nares). Fig. S1: User experience findings from the LFD Product Research Team. A Ease of use with nose and throat swabbing (N=54,415). B Recorded incidents of broken swabs with nose and throat swabbing (PCR and LFD). Fig. S2 Word cloud summary of d [file 44263_2024_121_MOESM1_ESM.docx]

***Supplementary Methods***

### ****NHSTT analysis****

To evaluate user attitudes towards nose and throat swabbing versus nose only swabbing, the LFD Product Research Team within NHSTT performed a post-hoc analysis of existing cross‑service user research as of April 2021. The existing research included testing surveys (of both service users and testing site leads), cross-service evaluation reports, and incident management reports related to swabbing. The objectives of this analysis were to determine if users have a preference for nose and throat swabbing versus nose only swabbing, and to estimate whether the implementation of nose only swabbing would increase uptake of testing (compared with nose and throat swabbing). The frequency of recurring themes raised by participants in existing research was assessed. From the testing survey results, the difference between proportions in swab preferences and swab-related incidents were calculated in R (version 4.3.2) using prop.test which implements a two-sided X^2^-test with continuity correction, as post-hoc analyses.

### User attitudes survey

Between March 4, 2021 and February 14, 2023, NHSTT conducted the Voice of the Customer (VOTC) program [1] which assessed attitudes towards SARS-CoV-2 testing among people in England based on their experience. The objective of the VOTC program was to capture, understand, and act on feedback to improve the testing service, and increase engagement and compliance. Data were collected at key touchpoints: test, trace, and isolation. The test survey captured feedback from people who had just taken a PCR test (at test sites or at home). Surveys were emailed using contact data collected as part of the COVID-19 National Testing Programme (stored within the National Pathology Exchange databases) and were completed by individuals online. The trace and isolate surveys captured feedback among adults in England who had just completed the contact tracing process (both cases and contacts) or had just completed their isolation period. Surveys were emailed using the Contact Tracing and Advisory Service database and were completed by individuals online. As part of these surveys, service users were asked the following question: "Please can you tell us why you are fairly likely or not sure if you would recommend getting a COVID-19 test?”. The collected answers to this question were assessed to determine those which included the description of detractors of SARS-CoV-2 testing. The free‑text descriptions for each of these detractors were then converted to text corpora using R (package: tm). Common words, so called stopwords, were removed based on the stopword lists from the Snowball stemmer project [2, 3]. Additionally, the words “test”, “done”, and “get” were removed. Multiple occurrences of a word within a single response were counted as one occurrence. The text corpora containing descriptions of detractors were analyzed to assess the number of occurrences of each detractor. This information was used to generate a “word cloud” of the most common detractors. The difference between proportions of certain detractors was also calculated in R (version 4.3.2) using prop.test as described above.

### Service evaluations

The service evaluations reported here were part of the wider program conducted in the UK from May 2020 to December 2021, some of which have been previously published [4-9]; an illustrative timeline is shown in Fig. 1. Aims and objectives of these service evaluation studies included: to determine whether LFD tests provided sufficient diagnostic performance for identifying SARS-CoV-2 (using the ‘gold standard’ of PCR as a reference) for use within the COVID-19 National Testing Programme [5, 7, 10]; to evaluate the performance of alternative swabbing techniques (e.g., nose only swabbing); to determine whether self-swabbing (SS) with LFDs was as effective when performed at home compared with a testing site; and to capture qualitative user feedback and insight on swabbing techniques. No performance benchmarks were set prior to the evaluations. For the purpose of this report, the studies have been named to include swabbing approach and test type, for example study *SS-AN-PCR1* refers to SS of anterior nares (AN) for PCR, study *AS-NT-LFD2* is the second study assessing assisted swabbing (AS) of the nose and throat (NT) for an LFD, and study *SS-MT-LFD1* refers to SS of both nostrils to mid-turbinate level (MT) for an LFD.

Methodology and device performance data for some of these service evaluations have been partially reported elsewhere as part of the UK DHSC’s commitment to evaluating the performance of in-vitro diagnostic devices for use in the COVID-19 National Testing Programme [4-9]. From the service evaluations reported here, six evaluated SS of anterior nares or both nostrils to mid-turbinate level for LFD or PCR testing (*SS-AN-PCR1*, *SS-AN-LFD1* to *SS-AN-LFD4*, and *SS-MT-LFD1*), three evaluated AS of both nose and throat and nose only (anterior nares) for LFDs (*AS-NT-LFD1*, *AS-AN-LFD1*, and *AS-AN-LFD2*), and three evaluated SS of the nose and throat for LFDs (*SS-NT-LFD1* to *SS-NT-LFD3*). Three studies (*SS-NT-LFD2*, *SS-AN-LFD3*, and *SS-AN-LFD4*) assessed the effectiveness of SS at users’ homes compared with at a designated testing site*.* Table 1 provides an overview of the service evaluations including start date, testing method, and swabbing technique can be found in. Additional information such as study ID and eligibility criteria are provided below and in the Supplementary Table 1.

**Eligibility criteria for the service evaluations**

- Individuals with (*AS-AN-LFD2*) or without symptoms of SARS-CoV-2 infection who have attended an NHS Test & Trace (NHSTT) regional or local test site for the purposes of receiving a diagnostic test (*all*)
- Participants were ≥18 years of age (*SS-AN-PCR1,* *AS-NT-LFD1*) or ≥16 years of age (*AS-AN-LFD1, AS-AN-LFD2, SS-NT-LFD2, SS-MT-LFD1, SS-AN-LFD3*)
- Participants were willing to self-collect throat and nose swab samples for the polymerase chain reaction (PCR) test (*SS-AN-PCR1, SS-NT-LFD1* to *SS-NT-LFD3, SS-AN-LFD1* to *SS-AN-LFD4,* and *SS-MT-LFD1),* or for children under 12 years of age the parent or guardian was willing to administer the swab on their behalf (*SS-NT-LFD1, SS-AN-LFD1*)
- Participants were willing to self-collect throat and nose (*SS-NT-LFD1* to *SS-NT-LFD3)* or double anterior nares (*SS-AN-PCR1, SS-AN-LFD1* to *SS-AN-LFD4*) or double mid-turbinate level (*SS-MT-LFD1*) swab samples for the lateral flow device (LFD) test (PCR test for *SS-AN-PCR1*)
- Participants agreed to take part in the assisted nose and throat, and nose only swabbing exercises (*AS-NT-LFD1, AS-AN-LFD1, AS-AN-LFD2*)
- Participants agreed to take part in the on-site sample collection and processing (*all*). For *SS-NT-LFD2, SS-AN-LFD3,* and *SS-AN-LFD4,* on-site sample collection was for PCR and participants agreed to take part in at-home sample collection and processing for their LFD test, and were willing to return the LFD result via digital systems within 4 hours of leaving the testing site
- Participants were willing to wait on-site for 15–30 minutes to receive their LFD result (*all except SS-AN-PCR1, SS-NT-LFD2, SS-AN-LFD3,* and *SS-AN-LFD4 as described above)* and understood the test result was indicative and may differ from the result of their diagnostic test (*all*)
- Participants consented to have the data from their PCR and/or LFD used (along with data collected as part of their test booking [*SS-NT-LFD3*]) as part of data capture/device validation exercise (*all*)
- Participants were willing to continue to self-isolate until they received their PCR result (*all*)
- A minimum of 50% of the cohort was to be composed of individuals who did not have experience of performing medical procedures (*SS-AN-PCR1*)

**Swab and assay specifics**

LFDs used in the service evaluations were the Innova SARS-Cov-2 Antigen Test (Xiamen Biotime Biotechnology, Fujian, China), the Orient Gene Covid-19 Ag Rapid Test Cassette (Zhejiang Orient Gene Biotech, Huzhou, China), and the SureScreen SARS-CoV-2 Antigen Rapid test Cassette (SureScreen Diagnostics, Nottingham, UK). These tests remained the same during the study period. Tests were performed according to the manufacturers’ instructions, including pre-specified interpretation of positive, negative, and void results. The process for assessing if LFD kits meet the standards set for inclusion in the COVID-19 National Testing Programme is outlined here: <https://www.gov.uk/>. Passing this standard is a requirement for inclusion in the procurement exercises undertaken, which are based on several criteria including cost.

Samples were collected at NHSTT regional and local testing sites. PCR testing was undertaken by laboratories within the NHSTT Lighthouse laboratory network. It was performed using either the ThermoFisher SARS-CoV-2 TaqPath COVID-19 CE-IVD qRT-PCR assay (ThermoFisher Scientific, Waltham, MA, USA), the Randox COVID‑19 qPCR kit (Randox Laboratories Ltd, Crumlin, County Antrim, United Kingdom), the Applied Biosystems TaqMan Fast Virus 1-step RT-PCR assay (ThermoFisher Scientific, Waltham, MA, USA), the PerkinElmer New Coronavirus Nucleic Acid Detection Kit (PerkinElmer Genomics, Pittsburgh, PA, USA), the PerkinElmer SARS-CoV-2 RT‑qPCR Reagent Kit (PerkinElmer Genomics, Pittsburgh, PA, USA), the Nonacus VirPath SARS-CoV-2 Multiplex qRT-PCR kit (Nonacus Ltd, Birmingham, UK) or the Clarigene SARS-CoV-2 assay (Yourgene Health, Manchester, UK) [4]. These assays amplify regions of the ORF1ab, N gene and S gene of the SARS-CoV-2 genome and use a bacteriophage MS2 as internal PCR and extraction control. qRT-PCR tests were classified as positive if a cycle threshold value (Ct) of less than 40 was observed for at least one of the three target genes [11]. Thresholds used to determine a positive PCR test were identical to those used for routine clinical reporting by each accredited laboratory [4].

### Observational data collection

Observational data collection for user experience was performed across the on-site service evaluations. Individuals who consented to participate in the service evaluations on-site were observed by trained study staff (observers) during swab collection and sample preparation and were asked to provide insight and feedback on the process. Participants performing SS were not assisted or guided with the sampling process, as this would not be representative of real-world use. Users were asked to rate how they found the swab or sample collection; answer options included: 1. Painful; 2. Uncomfortable; 3. Tickly; 4. Completely Painless; and 5. Blank. Observers were able to capture data on more than one user at a time, e.g., if there were a number of people testing within the same car at a drive-in testing site.

### Collection of quantitative data on testing outcomes

The methodology for the various service evaluations reported here was similar. All service evaluations compared an experimental testing device, method, or technique with a suitable control, and the same approach was undertaken for participants’ consent and data capture.

For studies evaluating SS, participants either swabbed their throat (both or bilateral peri-tonsillar areas) and a single nostril (to mid-turbinate level), hereafter referred to as nose and throat swabbing, and/or both anterior nares (shallow nose), according to the study objectives. In study *SS-MT-LFD1*, participants were also required to swab both their nostrils to mid-turbinate level. For studies evaluating AS, trained study staff swabbed the participant’s nose and throat, and/or anterior nares, according to the study objectives and standard diagnostic requirements.

For studies evaluating the effectiveness of SS LFDs, participants attending physical testing sites were required to process their own test sample using the LFD kit after reading the written instructions. While observers monitored the swabbing, they did not provide any guidance on the use of the LFD. After 15–30 minutes (depending on the type of LFD), participants interpreted the result of the LFD test themselves (positive, negative, or void) and these results were recorded by the trained observer. In the at-home studies, participants were required to collect the LFD at the regional testing site before returning home to take the test (ideally within 4 hours); participants were required to provide a photograph of the test result using the barcode and report the result. For studies evaluating the effectiveness of PCR, sample swabs were collected as per standard NHSTT instructions, sealed in a biohazard bag, and sent to an NHSTT network Lighthouse laboratory for PCR analysis. While on-site, the participant’s consent, barcodes for PCR sample(s), and/or LFD rapid test barcode(s) were captured and linked to the results. PCR test results were sent to participants by text message, as per standard NHSTT practice.

Viral concentration from the PCR tests was derived from cycle threshold (Ct) values using conversion formulae that were based on previous calibration in the respective laboratory. For calibration the Qnostics SCV2AQP01 quantitative SARS-CoV-2 standards panel was used resulting in an estimate of digital droplet PCR copies per mL [4, 12].

### Outcomes and statistical analyses

Matched LFD and qRT-PCR results (or two PCR samples in study *SS-AN-PCR1*) were analyzed to determine concordance, sensitivity (stratified by viral concentration), and specificity. Participants with missing data (e.g., for dropouts, samples not run, and void tests) were removed from all analyses except the void rate calculation. qRT-PCR-positive and -negative samples were used to analyze LFD sensitivity and specificity, respectively. The following definitions were used:

Sensitivity = TP/(TP+FN); Specificity = TN/(TN+FP), with true positives (TP) being where both LFD and PCR test were positive, true negatives (TP) being where both LFD and PCR test were negative, false positive (FP) being where LFD test was positive and PCR test was negative, and false negative (FN) being where LFD was negative and PCR was positive [11].

For the calculation of the 95% confidence intervals (CI) for sensitivity and specificity estimates, and for the observational data, the exact binomial CI method (Clopper-Pearson) was used. In the context of this study, we considered sufficient sensitivity for LFDs to be ≥50%, based on the threshold used for the original authorizations granted by the relevant regulatory body (Medicines and Healthcare products Regulatory Agency) [8].

Exploratory analyses compared the sensitivities of testing approaches between similar studies, based on differences in estimated sensitivities and overlapping 95% CIs. Post-hoc analyses assessing the differences of proportions in the observational data were calculated in R (version 4.3.2) using prop.test as described above.

Data for studies *AS-NT-LFD1* and *SS-NT-LFD1* have been published previously as part of initial investigations and validation of the Innova LFD and per NHSTT assurance [4-6, 8]*.* Similarly, data for *SS-NT-LFD2 and SS-MT-LFD1,* which were conducted when the Delta variant of concern was prominent, have been published as part of NHSTT assurance [7]. Other data from studies reported here have been included in larger data sets and assurance publications [4-9, 13]. These published data from the separate studies have been collated here to enable researchers to interpret the collective relevant information regarding the approaches taken to increase accessibility to testing. Some data are also represented here as part of the post-hoc indirect comparisons between swabbing techniques.

**Supplementary Results**

**Comparator assisted swabbing studies of nose and throat**

Study *AS-AN-LFD1* evaluated the effectiveness of AS of the nose and throat and anterior nares using the Orient Gene LFD versus PCR for identifying SARS-CoV-2. Overall, there were 2,598 evaluable paired PCR and LFD (nose and throat) samples and 2,597 evaluable paired PCR and LFD (anterior nares) samples. In the phase of the study assessing nose and throat swabbing using LFDs, 94% of samples were concordant for positive (*n*=232) or negative (*n*=2,204) results (Table S5). There was a statistically significant difference between discordant results in favor of PCR versus LFDs (*p*<0.001). Overall sensitivity of AS of the nose and throat with the Orient Gene LFD was 59.6% (vs. AS of the nose and throat using PCR; Table 1, Figure 3); sensitivity was 91.6% for samples with viral concentration >1M copies/mL (Table S6) [5]. Specificity was 99.8%.

**Comparator self-swabbing studies of nose and throat**

Study *SS-NT-LFD2* evaluated the effectiveness of SS of the nose and throat using the Innova LFD versus PCR for identifying SARS-CoV-2. All participants had the Delta variant of concern (VOC). Of these, 65.8% of participants with paired PCR and LFD samples were concordant for positive (Delta VOC) or negative results (Table S7). Overall sensitivity (Delta VOC) of SS of the nose and throat using the Innova LFD test was 65.8%; sensitivity was higher (81.4%) for samples with viral concentration >1M copies/mL (Table S8) [7]. Specificity was not available due to the data set consisting of positive PCR results only.

**Supplementary Tables**

### Table S1: Study ID of service evaluations

|  | **Study ID** |
| --- | --- |
| **AS vs. SS nose and throat studies** | |
| ***NT-PCR1* to *NT-PCR4* [14]** | COMBI006  COMBI021  COMBI031  COMBI045 |
| **SS nose only studies** | |
| ***SS-AN-PCR1*** | NOSE001 |
| ***SS-AN-LFD1*** | LFD008 |
| ***SS-AN-LFD2*** | LFD010 |
| ***SS-AN-LFD3*** | LFD015 |
| ***SS-AN-LFD4*** | LFD014 |
| ***SS-MT-LFD1*** | LFD016 |
| **SS nose and throat studies** |  |
| ***SS-NT-LFD1*** | LFD002 |
| ***SS-NT-LFD2*** | LFD017 |
| ***SS-NT-LFD3*** | LFD011 |
| **AS studies** |  |
| ***AS-AN-LFD1*** | LFD007 |
| ***AS-AN-LFD2*** | LFD018 |
| ***AS-NT-LFD1*** | LFD001 |

*NT-PCR1* to *NT-PCR4*: AS versus SS of the nose and throat for PCR. *SS-AN-PCR1*: SS of the anterior nares for PCR. *SS-AN-LFD1*: SS of the anterior nares using the Orient Gene LFD. *SS-AN-LFD2*: SS of anterior nares using the SureScreen LFD. *SS-AN-LFD3*: SS of anterior nares using the SureScreen LFD at participants’ homes. *SS-AN-LFD4*: SS of anterior nares using the Innova LFD at participants’ homes: *SS-MT-LFD1*: SS of both nostrils to mid-turbinate level using the Innova LFD. *SS-NT-LFD1* and *SS-NT-LFD2*: SS of the nose and throat using the Innova LFD. *SS-NT-LFD3*: SS of the nose and throat using the Innova LFD at participants’ homes. *AS-AN-LFD1:* AS of the anterior nares and/or nose and throat using the Orient Gene LFD. *AS-AN-LFD2:* AS of the anterior nares using the Innova LFD. *AS-NT-LFD1*: AS of the nose and throat using the Innova LFD.
AN, anterior nares; AS, assisted swabbing; LFD, lateral flow device; MT, mid-turbinate; NT, nose and throat; PCR, polymerase chain reaction; SS, self-swabbing.

**Table S2:** Numbers of void samples and other omitted samples in the service evaluations

|  | **Total number of void samples** | **Other omitted samples** |
| --- | --- | --- |
| **SS nose only studies** | | |
| ***SS-AN-PCR1*** | 60 (21 anterior nares, 29 nose and throat) | 7 incomplete pairs and 18 withdrawals |
| ***SS-AN-LFD1*** | 89 (56 PCR, 33 LFD)^b^ | N/A |
| ***SS-AN-LFD2*** | 103 (88 PCR, 15 LFD) | 19 withdrawals, 8 samples not run |
| ***SS-AN-LFD3*^a^** | 35 (34 PCR, one LFD) | N/A |
| ***SS-AN-LFD4* ^a^** | 102 (84 PCR, 18 LFD) | 28 individuals <16 years of age and one with incorrect age information; 203 user errors; 2,267 with incomplete or missing data or withdrew |
| ***SS-MT-LFD1*** | 44 (18 PCR, 26 LFD) | Eight individuals <16 years of age or no age provided; two withdrawals |
| **SS nose and throat studies** | | |
| ***SS-NT-LFD1*** | 87 (24 PCR, 63 LFD) | 36 testing kit failures; 27 user errors; 22 withdrawals |
| ***SS-NT-LFD2*** | Three (all LFD) | One withdrawal |
| ***SS-NT-LFD3* ^a^** | 19 (all PCR) | 93 submitted after 1 day, 10 results not included, 52 unable to be paired, 10 lab results outstanding |
| **AS studies** | | |
| ***AS-AN-LFD1*** | 31 (19 PCR, 12 anterior nares LFD, eight nose and throat LFD) | Four with incomplete data; seven with no LFD anterior nares sample and two with no LFD nose and throat sample |
| ***AS-AN-LFD2*** | 15 (eight PCR, seven LFD) | 46 with missing or incomplete data; 10 <16 years of age |
| ***AS-NT-LFD1*** | 57 (40 PCR, 17 LFD) | 74 due to processing at unconfirmed labs |

^a^Effectiveness of LFD tested at participants’ homes. ^b^One paired sample was void for both PCR and LFD

AN, anterior nares; AS, assisted swabbing; LFD, lateral flow device; N/A, not applicable; NT, nose and throat; PCR, polymerase chain reaction; SS, self-swabbing.

**Table S3:** *SS-AN-PCR1*: Concordance matrix of nose and throat swabbing versus nose only (anterior nares) swabbing

|  | **Nose and throat swabbing** | | | |
| --- | --- | --- | --- | --- |
|  | **Overall** | **Positive** | **Negative** | **Sum** |
| **Nose only swabbing** | **Positive** | 341 | 15 | 356 |
|  | **Negative** | 46 | 1,681 | 1727 |
|  | **Sum** | 387 | 1696 | 2083 |

*SS-AN-PCR1:* SS of anterior nares versus nose and throat using PCR.

AN, anterior nares; PCR, polymerase chain reaction; SS, self-swabbing.

**Table S4:** *SS-AN-PCR1* (anterior nares): quantitative data analysis by Ct

|  |  | **Nose and throat swabbing** | | | |
| --- | --- | --- | --- | --- | --- |
|  | **Ct group** | **Higher VC (Ct <25)** | **Lower VC (Ct ≥25 and <35)** | **Negative** | **Total** |
| **Nose only swabbing** | **Higher VC (Ct <25)** | 228 | **11** | **2** | 241 |
|  | **Lower VC (Ct ≥25 and <35)** | **39** | 58 | **12** | 109 |
|  | **Negative** | **3** | **42** | 1,688 | 1,733 |
|  | **Total** | 270 | 111 | 1,702 | 2,083 |

A total of 84 samples showed higher concentrations for the nose and throat swabbing and 25 samples showed higher concentrations for nose only swabbing. qRT-PCR tests were classified as positive if Ct <40 was observed for at least one of the three target genes.

*SS-AN-PCR1*: SS of anterior nares versus nose and throat using PCR.

AN, anterior nares; Ct, cycle threshold; PCR, polymerase chain reaction; SS, self-swabbing; VC, viral concentration.

**Table S5:** Concordance matrix of LFD and PCR results in assisted-swabbing studies: *AS-NT-LFD1* (nose and throat), *AS-AN-LFD1* (anterior nares), and *AS-AN-LFD2* (anterior nares).

|  |  | **qRT-PCR** | | |
| --- | --- | --- | --- | --- |
|  |  | **Positive** | **Negative** | **Sum** |
| ***AS-NT-LFD1*** Nose and throat | **Positive** | 376 | 15 | 391 |
|  | **Negative** | 315 | 3,519 | 3,834 |
|  | **Sum** | 691 | 3,534 | 4,225 |
|  |  |  |  |  |
| ***AS-AN-LFD1*** Nose and throat | **Positive** | 232 | 5 | 237 |
|  | **Negative** | 157 | 2,204 | 2,361 |
|  | **Sum** | 389 | 2,209 | 2,598 |
|  |  |  |  |  |
| ***AS-AN-LFD1*** Anterior nares | **Positive** | 207 | 4 | 211 |
|  | **Negative** | 182 | 2,204 | 2,386 |
|  | **Sum** | 389 | 2,208 | 2,597 |
|  |  |  |  |  |
| ***AS-AN-LFD2*** Anterior nares | **Positive** | 163 | 5 | 168 |
|  | **Negative** | 120 | 872 | 992 |
|  | **Sum** | 283 | 877 | 1,160 |

*AS-NT-LFD1*: AS of the nose and throat using the Innova LFD. *AS-AN-LFD1:* AS of the anterior nares and/ nose and throat using the Orient Gene LFD. *AS-AN-LFD2:* AS of the anterior nares using the Innova LFD.
**Green** shaded cells represent concordant results. **Blue** shaded cells represent the total number of paired samples.

AN, anterior nares; AS, assisted swabbing; LFD, lateral flow device; PCR, polymerase chain reaction; qRT-PCR, quantitative reverse transcription polymerase chain reaction; NT, nose and throat.

**Table S6:** Sensitivity of assisted swabbing using LFDs overall and by viral concentration, compared with assisted swabbing of the nose and throat with PCR, in studies *AS-NT-LFD1* (nose and throat), *AS-AN-LFD1* (anterior nares), and *AS-AN-LFD2* (anterior nares).

|  | **Sensitivity, % (95% CI)** | | | |
| --- | --- | --- | --- | --- |
|  | **Viral concentration** | | | **Overall** |
|  | **>1M** | **10K**–**1M** | **<10K** |  |
| ***AS-NT-LFD1* [5]** Nose and throat | 84.5  (79.3–88.9) | 56.5  (50.4–62.5) | 12.6 (8.1–18.4) | 54.4 (50.6–58.2) |
| ***AS-AN-LFD1*** Nose and throat | 91.6 (86.1–95.5) | 59.1 (50.4–67.4) | 9.3 (4.3– 16.9) | 59.6 (54.6–64.6) |
| ***AS-AN-LFD1* [5]** Anterior nares | 88.3 (82.2–92.9) | 47.1 (38.5–55.8) | 7.1 (2.9–14.0) | 53.2  (48.1–58.3) |
| ***AS-AN-LFD2*** Anterior nares | 89.5  (80.3–95.3) | 58.3  (49.4–66.9) | 24.0  (14.9–35.3) | 57.6 (51.6–63.4) |

*AS-NT-LFD1*: AS of the nose and throat using the Innova LFD. *AS-AN-LFD1:* AS of the anterior nares or and nose and throat using the Orient Gene LFD. *AS-AN-LFD2:* AS of the anterior nares using the Innova LFD.
AN, anterior nares; AS, assisted swabbing; CI, confidence interval; LFD, lateral flow device; NT, nose and throat; PCR, polymerase chain reaction.

**Table S7:** Concordance matrix of LFD and PCR results in Studies *SS-NT-LFD1* (nose and throat), *SS-NT-LFD2* (nose and throat), *SS-AN-LFD1* (anterior nares), *SS-AN-LFD2* (anterior nares), and *SS-MT-LFD1* (mid-turbinate level).

|  |  | **qRT-PCR** | | |
| --- | --- | --- | --- | --- |
|  |  | **Positive** | **Negative** | **Sum** |
| ***SS-NT-LFD1*** Nose and throat | **Positive** | 212 | 19 | 231 |
|  | **Negative** | 212 | 2,030 | 2,242 |
|  | **Sum** | 424 | 2,039 | 2,473 |
|  |  |  |  |  |
| ***SS-NT-LFD2*** Nose and throat | **Positive** | 415 | 0 | 415 |
|  | **Negative** | 216 | 0 | 216 |
|  | **Sum** | 631 | 0 | 631 |
|  |  |  |  |  |
| ***SS-AN-LFD1*** Anterior nares | **Positive** | 204 | 10 | 214 |
|  | **Negative** | 179 | 1,395 | 1,574 |
|  | **Sum** | 383 | 1,405 | 1,788 |
|  |  |  |  |  |
| ***SS-AN-LFD2*** Anterior nares | **Positive** | 215 | 0 | 215 |
|  | **Negative** | 88 | 2,091 | 2,179 |
|  | **Sum** | 303 | 2,091 | 2,394 |
|  |  |  |  |  |
| ***SS-MT-LFD1*** Mid-turbinate level | **Positive** | 237 | 2 | 239 |
|  | **Negative** | 88 | 775 | 863 |
|  | **Sum** | 325 | 777 | 1,102 |

*SS-NT-LFD1*: SS of the nose and throat using the Innova LFD. *SS-NT-LFD2:* SS of the nose and throat using the Innova LFD. *SS-AN-LFD1:* SS of the anterior nares using the Orient Gene LFD. *SS-AN-LFD2:* SS of the anterior nares using the SureScreen LFD. *SS-MT-LFD1:* SS of both nostrils to mid-turbinate level using the Innova LFD.
**Green** shaded cells represent concordant results. **Blue** shaded cells represent the total number of paired samples.
AN, anterior nares, LFD, lateral flow device; MT, mid-turbinate; NT, nose and throat; PCR, polymerase chain reaction; qRT-PCR, quantitative reverse transcription polymerase chain reaction; SS, self-swabbing

**Table S8:** Sensitivity of self-swabbing using LFDs overall and by viral concentration, compared with self-swabbing of the nose and throat with PCR, in Studies *SS-NT-LFD1* (nose and throat), *SS-AN-LFD1* (anterior nares), *SS-AN-LFD2* (anterior nares), *SS-NT-LFD2* (nose and throat), and *SS-MT-LFD1* (mid-turbinate level).

|  | **Sensitivity, % (95% CI)** | | | |
| --- | --- | --- | --- | --- |
|  | **Viral concentration** | | | **Overall** |
|  | **>1M** | **10K**–**1M** | **<10K** |  |
| ***SS-NT-LFD1*** Nose and throat | 80.5 (72.2–87.2) | 54.5 (47.1–61.7) | 12.0 (6.7–19.3) | 50.0 (45.1–54.9) |
| ***SS-NT-LFD2 [7]*** Nose and throat | 81.4  (76.9–85.3) | 54.6  (47.7–61.4) | 19.7  (10.9–31.3) | 65.8 (61.9–69.5) |
| ***SS-AN-LFD1* [5]** Anterior nares | 87.8  (81.1–92.7) | 53.4  (44.5–62.2) | 10.6  (5.6–17.8) | 53.3  (48.1–58.4) |
| ***SS-AN-LFD2*** Anterior nares | 95.2  (89.8–98.2) | 73.3  (63.8–81.5) | 27.1  (17.2–39.1) | 71.0  (65.5–76.0) |
| ***SS-MT-LFD1 [7]*** Mid-turbinate | 98.1 (94.4–99.6) | 63.6 (53.9–72.6) | 18.8 (9.0–32.6) | 72.9  (67.7–77.7) |

*SS-NT-LFD1*: SS of the nose and throat using the Innova LFD. *SS-NT-LFD2:* SS of the nose and throat using the Innova LFD (Delta variant of concern). *SS-AN-LFD1:* SS of anterior nares using Orient Gene LFD. *SS-AN-LFD2:* SS of the anterior nares using the SureScreen LFD. *SS-MT-LFD1:* SS of both nostrils to mid-turbinate level using the Innova LFD.

AN, anterior nares; CI, confidence interval; LFD, lateral flow device; MT, mid-turbinate; NT, nose and throat; PCR, polymerase chain reaction; SS, self-swabbing.

**Table S9:** Concordance matrix of LFD and PCR results in Studies *SS-NT-LFD3* (nose and throat), *SS-AN-LFD3* (anterior nares), and *SS-AN-LFD4* (anterior nares)

|  |  | **qRT-PCR** | | |
| --- | --- | --- | --- | --- |
|  |  | **Positive** | **Negative** | **Sum** |
| ***SS-NT-LFD3*** Nose and throat | **Positive** | 46 | 2 | 48 |
|  | **Negative** | 35 | 690 | 725 |
|  | **Sum** | 81 | 692 | 773 |
|  |  |  |  |  |
| ***SS-AN-LFD3*** Anterior nares | **Positive** | 272 | 4 | 276 |
|  | **Negative** | 92 | 1,131 | 1,223 |
|  | **Sum** | 364 | 1,135 | 1,499 |
|  |  |  |  |  |
| ***SS-AN-LFD4*** Anterior nares | **Positive** | 486 | 26 | 512 |
|  | **Negative** | 163 | 3,315 | 3,478 |
|  | **Sum** | 649 | 3,341 | 3,990 |

*SS-NT-LFD3*: SS of the nose and throat using the Innova LFD at participants’ homes. *SS‑AN-LFD3*: SS of the anterior nares using the SureScreen LFD at participants’ homes. *SS-AN-LFD4*: SS of the anterior nares using the Innova LFD at participants’ homes. **Green** shaded cells represent concordant results. **Blue** shaded cells represent the total number of paired samples.
AN, anterior nares; LFD, lateral flow device; NT, nose and throat; PCR, polymerase chain reaction; qRT-PCR, quantitative reverse transcription polymerase chain reaction.

### Table S10: Sensitivity of at-home self-swabbing using LFDs overall and by viral concentration, compared with self-swabbing of the nose and throat with PCR, in Studies SS-NT-LFD3 (nose and throat), SS-AN-LFD3 (anterior nares), and SS-AN-LFD4 (anterior nares)

|  | **Sensitivity, % (95% CI)** | | | |
| --- | --- | --- | --- | --- |
|  | **Viral concentration** | | | **Overall** |
|  | **>1M** | **10K–1M** | **<10K** |  |
| ***SS-NT-LFD3*** Nose and throat | 90.6 (80.5–100) | 60.0 (40.8–79.2) | 8.3 (0.0–19.4) | 56.8 (44.3–67.8) |
| ***SS-AN-LFD3*** Anterior nares | 93.3  (88.8–96.4) | 68.4 (59.1–76.7) | 22.2  (12.0–35.6) | 74.7  (69.9–79.1) |
| ***SS-AN-LFD4*** Anterior nares | 97.6  (95.0–99.0) | 75.1 (69.0–80.6) | 22.1 (15.1–30.5) | 74.9 (71.4–78.2) |

*SS-NT-LFD3*: SS of the nose and throat using the Innova LFD at participants’ homes. *SS‑AN-LFD3:* SS of the anterior nares using the SureScreen LFD performed at participants’ homes. *SS-AN-LFD4*: SS of the anterior nares using the Innova LFD at participants’ homes.
AN, anterior nares; CI, confidence interval; LFD, lateral flow device; NT, nose and throat; PCR, polymerase chain reaction; SS, self-swabbing.

**Supplementary Figures**

**Fig. S1**


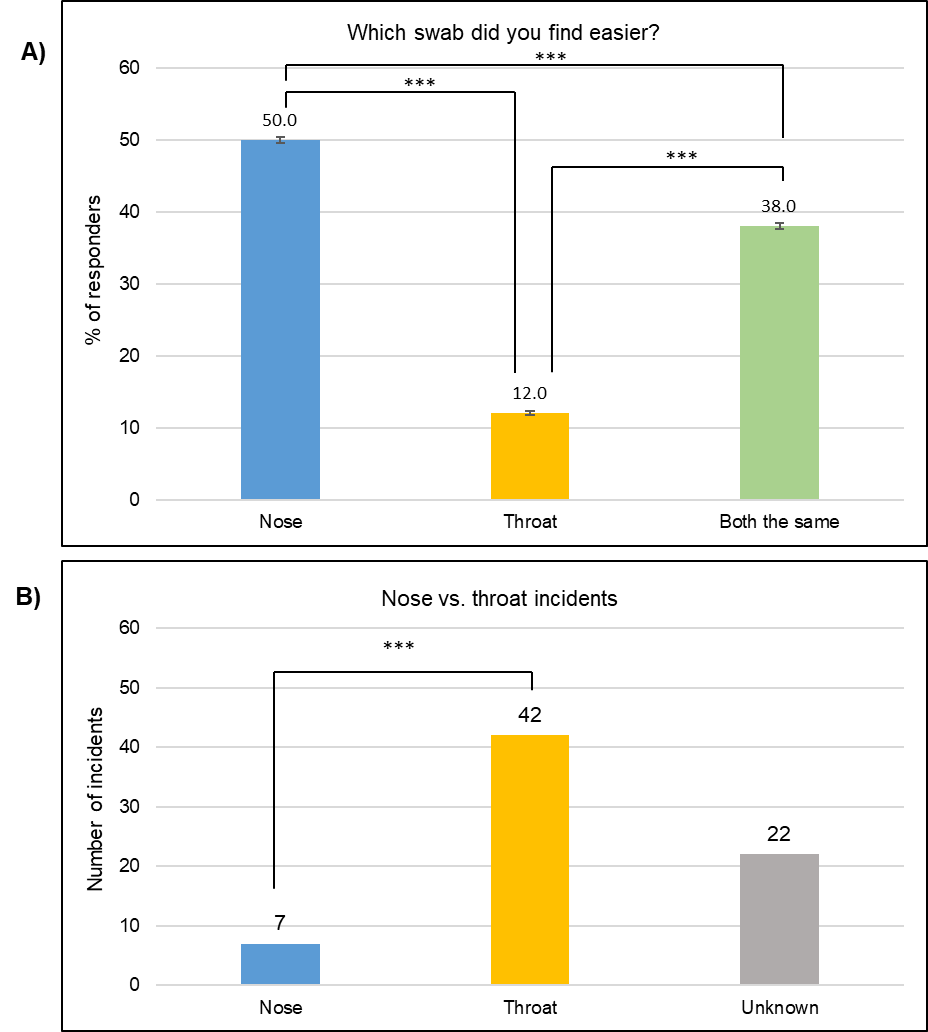


**User experience findings from the LFD Product Research Team.**

**A** Ease of use with nose and throat swabbing (*N*=54,415). **B** Recorded incidents of broken swabs with nose and throat swabbing (PCR and LFD)

****p*<0.0001. p values derived from chi-square tests comparing nose vs. throat swabbing.

**Panel A:** Data are mean and 95% confidence intervals.

LFD, lateral flow device; PCR, polymerase chain reaction.

**Fig. S2**


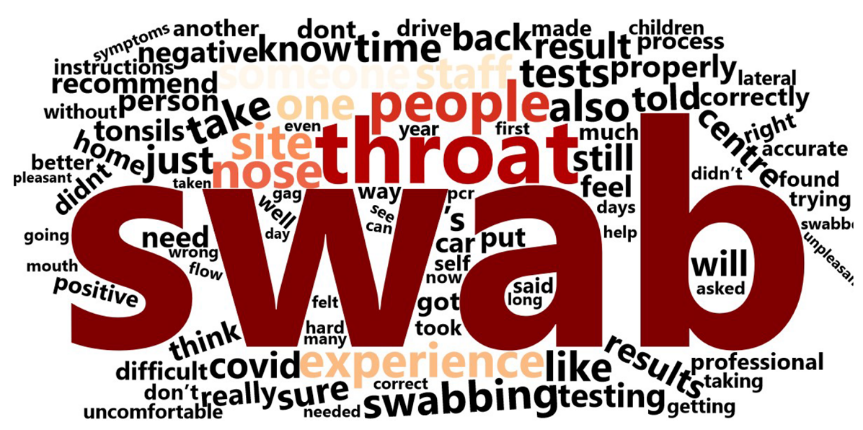

**Word cloud summary of detractor descriptions for SARS-CoV-2 swabbing**

Answers provided by the UK general public were in response to the question: "Please can you tell us why you are fairly likely or not sure if you would recommend getting a COVID-19 test?” Larger size of detractors represents a higher incidence in responses.

**References**

1. **COVID-19: general public testing behaviours** [<https://www.gov.uk/government/publications/lfd-tests-how-and-why-they-were-used-during-the-pandemic/covid-19-general-public-testing-behaviours>]

2. **snowball stopword list v2.3** [<https://search.r-project.org/CRAN/refmans/stopwords/html/data_stopwords_snowball.html>]

3. **Snowball** [<https://snowballstem.org/>]

4. Eyre DW, Futschik M, Tunkel S, Wei J, Cole-Hamilton J, Saquib R, Germanacos N, Dodgson AR, Klapper PE, Sudhanva M *et al*: **Performance of antigen lateral flow devices in the UK during the alpha, delta, and omicron waves of the SARS-CoV-2 pandemic: a diagnostic and observational study**. *Lancet Infect Dis* 2023, **23**(8):922-932.

5. **Asymptomatic testing for SARSCoV-2 using antigen-detecting lateral flow devices** [<https://assets.publishing.service.gov.uk/media/60e56b3ee90e0764c6eb3866/asymptomatic-testing-for-SARS-CoV-2-using-antigen-detecting-lateral-flow-devices-evidence-from-performance-data-Oct-2020-to-May-2021.pdf>]

6. Futschik ME, Johnson S, Turek E, Chapman D, Carr S, Thorlu-Bangura Z, Klapper PE, Sudhanva M, Dodgson A, Cole-Hamilton JR *et al*: **Rapid antigen testing for SARS-CoV-2 by lateral flow assay: a field evaluation of self- and professional testing at UK community testing sites**. *J Clin Virol* 2024, **171**:105654.

7. **Testing for SARS-CoV-2 using antigen detecting lateral flow devices. Evidence from performance assessment and evaluation for the period February 2021 to March 2022** [<https://assets.publishing.service.gov.uk/media/6416dc91d3bf7f79d9675d96/SARS-CoV-2-testing-using-LFD-evidence-from-performance-data-part-2.pdf>]

8. **Evaluation of lateral flow device performance within the National Testing Programme: Date of reporting period: 8 November 2020 to 21 March 2022** [<https://assets.publishing.service.gov.uk/media/638a1ff18fa8f569f4902038/LFD_Performance_within_the_NTP_Report.pdf>]

9. **Research and analysis: Lateral flow device (LFD) performance data** [<https://www.gov.uk/government/publications/lateral-flow-device-performance-data>]

10. Peto T: **COVID-19: rapid antigen detection for SARS-CoV-2 by lateral flow assay: a national systematic evaluation of sensitivity and specificity for mass-testing**. *EClinicalMedicine* 2021, **36**:100924.

11. Futschik ME, Johnson S, Turek E, Chapman D, Carr S, Thorlu-Bangura Z, Klapper PE, Sudhanva M, Dodgson A, Cole-Hamilton JR *et al*: **Rapid antigen testing for SARS-CoV-2 by lateral flow assay: A field evaluation of self- and professional testing at UK community testing sites**. *Journal of clinical virology : the official publication of the Pan American Society for Clinical Virology* 2024, **171**:105654.

12. **PCR testing for SARS-CoV-2 during the COVID-19 pandemic** [<https://www.gov.uk/government/publications/pcr-testing-for-sars-cov-2-during-the-covid-19-pandemic>]

13. **Technical Report: In vitro and clinical post-market surveillance of Biotime SARS-CoV-2 Lateral Flow Antigen Device in detecting the SARS-CoV-2 Delta variant (B.1.617.2)** [<https://assets.publishing.service.gov.uk/media/60e56bf4e90e0764ccfbd6bf/in-vitro-and-clinical-post-market-surveillance-of-Biotime-SARS-CoV-2-Lateral-Flow-Antigen-Device-in-detecting-the-SARS-CoV-2-Delta-variant-B.1.617.2.pdf>]

14. Fowler T, Chapman D, Futschik ME, Tunkel SA, Blandford E, Turek E, Kolade O, da Cunha SS, Dodgson A, Klapper P *et al*: **Self-swabbing versus assisted swabbing for viral detection by qRT-PCR: the experience from SARS-CoV-2 based on a meta-analysis of six prospectively designed evaluations conducted in a UK setting**. *Eur J Clin Microbiol Infect Dis* 2024, **43**(8):1621-1630.
